# Association of short-term blood pressure variability with cardiovascular mortality among incident hemodialysis patients

**DOI:** 10.1080/0886022X.2018.1456456

**Published:** 2018-04-05

**Authors:** Yiduo Feng, Ziqian Li, Jing Liu, Fang Sun, Lijie Ma, Yang Shen, Yilun Zhou

**Affiliations:** a Department of Nephrology, Beijing Tiantan Hospital, Capital Medical University, Beijing, China;; b Department of Clinical Nutrition, Peking University First Hospital, Beijing, China;; c Department of Nephrology, Beijing Chaoyang Hospital, Capital Medical University, Beijing, China

**Keywords:** Hemodialysis, blood pressure variability, cardiovascular mortality

## Abstract

**Objective:** To investigate the association of short-term blood pressure variability (BPV) with cardiovascular mortality in hemodialysis (HD) patients, using a reliable index called average real variability (ARV), and to assess the factors associated with ARV in incident HD population.

**Methods:** A total of 103 HD patients were recruited, with 44-h ambulatory blood pressure monitoring performed after the midweek HD session. Systolic BPV was assessed by *SD*, coefficient of variation (CV), and ARV, respectively. Laboratory data were obtained from blood samples before the midweek HD. All patients were followed up for 24 months.

**Results:** According to the median of BPV indices, the comparisons between patients with the low and high values were conducted. Kaplan–Meier analysis showed the survival curves corresponding to median of *SD* and CV exhibit similar performance for the low and high groups (*p* = .647, *p* = .098, respectively). In contrast, patients with higher ARV had a lower survival rate than those with lower ARV (77.8% vs. 98.0%, *p* = .002). After adjustment for demographics and clinical factors, ARV (HR: 1.143; 95% CI: 1.022–1.279, *p* = .019) and high-sensitivity C-reactive protein (HR: 1.394; 95% CI: 1.025–1.363, *p* = .021) were associated with increased risk of cardiovascular mortality in HD patients. Age and interdialytic weight gain (IDWG) were related factors for ARV (β = 0.065, *p* = .005; β = 0.825, *p* = .003, respectively).

**Conclusions:** Greater ARV was independently associated with increased risk of cardiovascular mortality in HD patients. Age and IDWG were independent related factors for ARV.

## Introduction

End stage renal disease (ESRD) is associated with a 3- to 10-fold increased risk of cardiovascular events as compared with general populations [1,2]. Cardiac disease is the leading cause of death among patients with ESRD, representing half of all-cause mortality [3,4]. Hypertension may contribute to the observed excess in cardiovascular disease. However, blood pressure (BP) per se, may not fully characterize the pathophysiologic relationship between BP and cardiovascular outcomes. Other factors, such as blood pressure variability (BPV), are also prognostically important. Existing data linking BPV from ambulatory blood pressure monitoring (ABPM) with all-cause mortality in the general population are robust [5–7]. In hemodialysis (HD) population, ambulatory BP has been demonstrated to be the best predictor of all-cause mortality in a long-term follow-up study compared with pre- or post-dialysis BP [8]. However, sparse data are available regarding the association of ambulatory BPV with outcomes in dialysis patients.

Recently, a reliable index inspired by the total variability concept of real analysis in mathematics called average real variability (ARV) has been indicated for establishing the prognostic significance of BPV [9]. ARV is a more reliable representation of time series variability than *SD* and may be less sensitive to the relative low sampling frequency of the ABPM devices in general people. However, no data are available concerning about ARV in patients on HD.

The purposes of this study are to investigate the association of short-term BPV assessed by ARV and other traditional indices with cardiovascular mortality in HD patients and to analyze the risk factors associated with higher ARV in incident HD population.

## Methods

### Subjects

HD patients were recruited when: (1) they had been on HD for more than 3 months; (2) they had no clinical cardiovascular disease during 3 months preceding entry into the study; (3) they had no known acute inflammatory event, malignant disease, and the serum albumin >30 g/L; (4) they had no chronic liver disease and chronic obstructive pulmonary disease; and (5) they had no pitting edema in either leg before HD. In all, 103 patients fulfilled the entry criteria. During the follow-up period, all patients were dialyzed 3 times weekly and 4 h for each treatment session, with polysulfone hollow-fiber dialyzer (F7, Fresenius Medical Care AG). Informed consent was obtained from every participant and the study protocol was approved by the ethics committee of the center.

### Study design

All patients had 44-h ambulatory BP monitoring performed after the midweek dialysis. Demographic and medical data including age, gender, smoking history, and comorbid conditions were obtained from medical records and by patient interviews. Laboratory data were obtained from fasting blood samples before the midweek HD. All patients recruited were followed up for 24 months. The occurrence of cardiovascular deaths was recorded. Cardiovascular deaths were confirmed and ascertained from medical records by two cardiologists, with disagreements resolved by a third cardiologist.

### Measures

#### Ambulatory BP monitoring

All subjects underwent 44 h ABPM with a fully automatic device that met the criteria of the Association for the Advancement of Medical Instrumentation. Ambulatory BPs and heart rates were recorded every 20 min during the day (6 am to 10 pm), and every 30 min during the night (10 pm to 6 am). Participants were told to carry on with their normal daily activities during measurements. Patients with less than 80% valid measurements were excluded. These data were exported to a relational database to allow for data management as well as centering the time to that elapsed after dialysis with standard programming tools.

#### BPV metric

Systolic BP is a major contributor to pulse pressure and is generally confirmed as the risk factor for mortality in dialysis people. We therefore decided to assess the factors associated with systolic BPV and quantified the association of systolic BPV with cardiovascular mortality in HD patients.

We described 44-h systolic BPV using *SD*, coefficient of variation (CV) (*SD*/mean) and ARV. ARV was calculated using the following formula:

$$\text{ARV}=\frac{1}{N-1}\sum_{k=1}^{N-1}\left|BP_{k+1}-BP_{k}\right|$$

where *N* denotes the number of valid systolic BP measurements corresponding to a given subject.

### Statistical analyses

Data were analyzed using Statistical Product and Service Solutions (SPSS) version 19.0. Data were expressed as mean/*SD* or median (25th–75th percentile) values for continuous variables and as frequency distributions for categorical variables. Statistical significance of the differences between groups was tested using *t*-test or Wilcoxon rank-sum test for continuous variables and χ^2^ test for categorical variables. We used receiver operating characteristic (ROC) curve to determine the prognostic ability of BPV. Multivariable logistic regression analysis was used to identify the factors associated with BPV. The prognostic value was determined using Cox proportional hazard analysis. The proportionality assumption was tested and confirmed by Schoenfeld residual testing. The association with cardiovascular mortality was measured using Kaplan–Meier analysis and log-rank testing. *p* < .05 was considered to be significant.

## Results

### Basic characters of all dialysis patients

A total of 103 patients were recruited in this study. Five people accepted renal transportation and one person transferred to another hospital during follow-up period. The characteristics of these patients at baseline were listed in Table 1. The average age was 57.9 ± 12.77 years, 45 patients were chronic glomerulonephritis, 23 patients were diabetes, 18 patients were primary hypertension, 10 patients were interstitial nephritis, and 7 patients were unknown.

**Table 1.** Baseline characteristics of participants.

Characteristics	All patients (*n* = 103)
Age (years)	57.49 ± 12.77
Sex (% men)	60 (58.25)
Body mass index (kg/m^2^)	23.31 (20.22, 24.96)
Hemodialysis vintage (months)	68.39 ± 42.80
History of diabetes (%)	23 (22.33)
Laboratory values	
Hemoglobin (g/L)	123.32 ± 11.78
Albumin (g/L)	37.46 ± 2.20
Prealbumin (g/L)	0.47 ± 0.09
Triglyceride (mmol/L)	1.93 ± 1.07
Total cholesterol (mmol/L)	4.44 ± 0.92
HDL-cholesterol (mmol/L)	0.93 ± 0.43
LDL-cholesterol (mmol/L)	2.31 ± 0.70
Calcium (mmol/L)	2.24 ± 0.18
Phosphorous (mmol/L)	1.82 ± 0.54
Hs-CRP (mg/L)	1.96 (1.11, 4.36)
Ferritin (ng/mL)	634.29 ± 367.95
iPTH (pg/ml)	250.28 (125.50, 445.70)
Ambulatory blood pressure monitoring	
44-h SBP (mmHg)	157.09 ± 16.25
*SD* of 44-h SBP (mmHg)	20.06 ± 5.31
CV of 44-h SBP (%)	12.94 ± 3.76
ARV of 44-h SBP (mmHg)	18.26 ± 4.84

HDL: high-density lipoprotein; LDL: low-density lipoprotein; Hs-CRP: high-sensitivity C-reactive protein; iPTH: intact parathyroid hormone; SBP: systolic blood pressure; CV: coefficient of variation; ARV: average real variability.

### Associations between BPV and cardiovascular mortality

During the follow-up period, 12 deaths were recorded, including 11 due to cardiovascular causes and 1 due to infectious disease.

**Figure 1.**
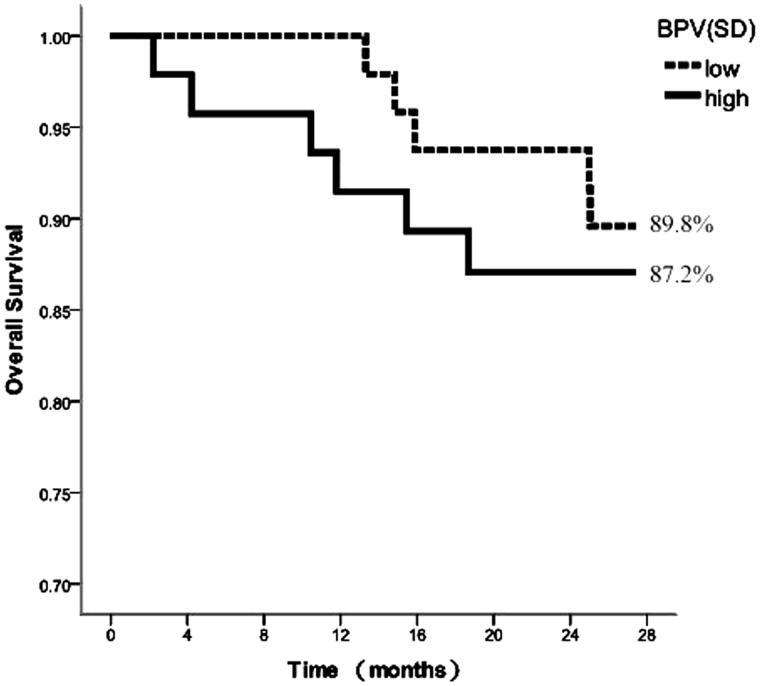
Kaplan–Meier survival curves for cardiovascular mortality according to median of 44-h SBPSD.

**Figure 2.**
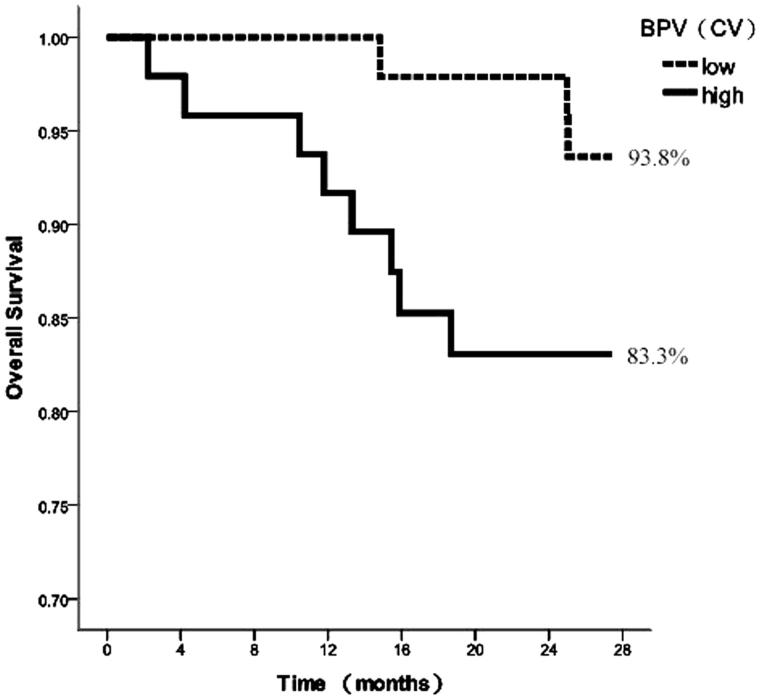
Kaplan–Meier survival curves for cardiovascular mortality according to median of 44-h SBPCV.

**Figure 3.**
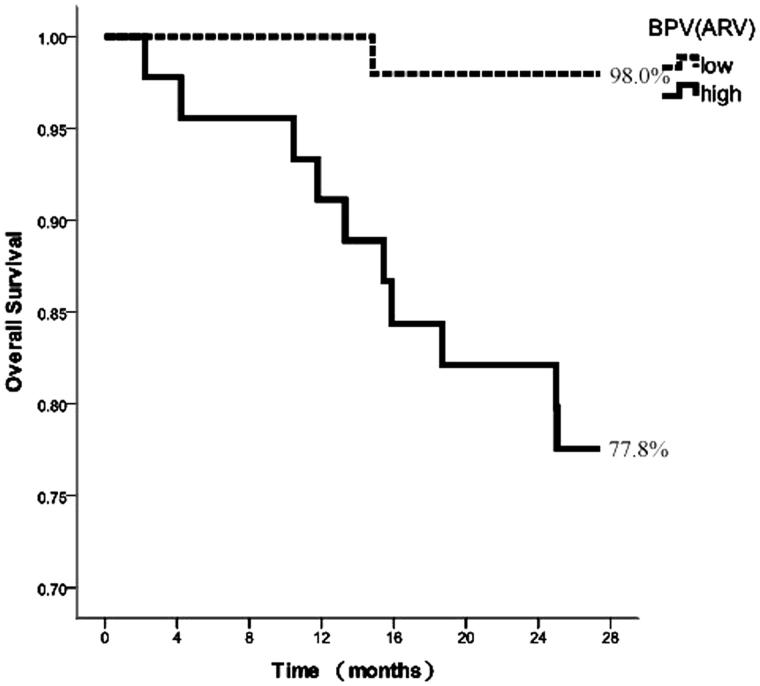
Kaplan–Meier survival curves for cardiovascular mortality according to median of ARV.

According to the median of BPV indices, the comparisons between patients with the low and high values were conducted. Figures 1–3 depicted the survival curves calculated using the Kaplan–Meier method for the aforementioned groups considering *SD*, CV, and ARV as variability indices, respectively.

The survival curves corresponding to *SD* exhibited similar performance for the high and low *SD* groups, with no significant difference in survival rates (87.2% vs. 89.8%), assessed by the log-rank test (*χ*^2^ = 0.210, *p* = .647, Figure 1). The survival rates of high CV were lower than those of low CV (83.3% vs. 93.8%), although the survival curves showed no significant difference, as determined by the log-rank test (*χ*^2^ = 2.738, *p* = .098, Figure 2). In contrast, the survival curves corresponding to the ARV index were statistically different, lower ARV was associated with improved survival (77.8% vs. 98.0%). The log-rank test showed that the risks among the groups were significantly different (*χ*^2^ = 9.645, *p* = .002, Figure 3).

In multivariable cox analysis, higher ARV and high-sensitivity C-reactive protein (hs-CRP) level were associated with an increased risk of cardiovascular mortality both in minimally and fully adjusted models (*p* < .05) (Table 2). Age was not involved in Cox analysis because ARV was positively correlated with age (Pearson’s correlation coefficients were 0.471).

**Table 2.** Risk factors for cardiovascular mortality in hemodialysis patients using cox regression analysis.

	Unadjusted		Minimally adjusted^a^		Fully adjusted^b^	
Parameters	HR (95%CI)	*p*	HR (95%CI)	*p*	HR (95%CI)	*p*
ARV	1.157 (1.057–1.266)	.002	1.130 (1.020–1.252)	.019	1.143 (1.022–1.279)	.019
Hs-CRP	1.230 (1.092–1.386)	.001	1.178 (1.042–1.332)	.009	1.394 (1.025–1.363)	.021

Values expressed as hazard ratios (HR) and 95% confidence interval (CI).

Hs-CRP: high-sensitivity C-reactive protein; SBP: systolic blood pressure; ARV: average real variability.

^a^Adjusted for ARV, hs-CRP, 44-h systolic blood pressure (44-h SBP) and phosphate level.

^b^Adjusted for sex, dialysis vintage, BMI, IDWT, albumin level, ARV, hs-CRP, 44-h systolic blood pressure (44-h SBP) and phosphate level.

**Figure 4.**
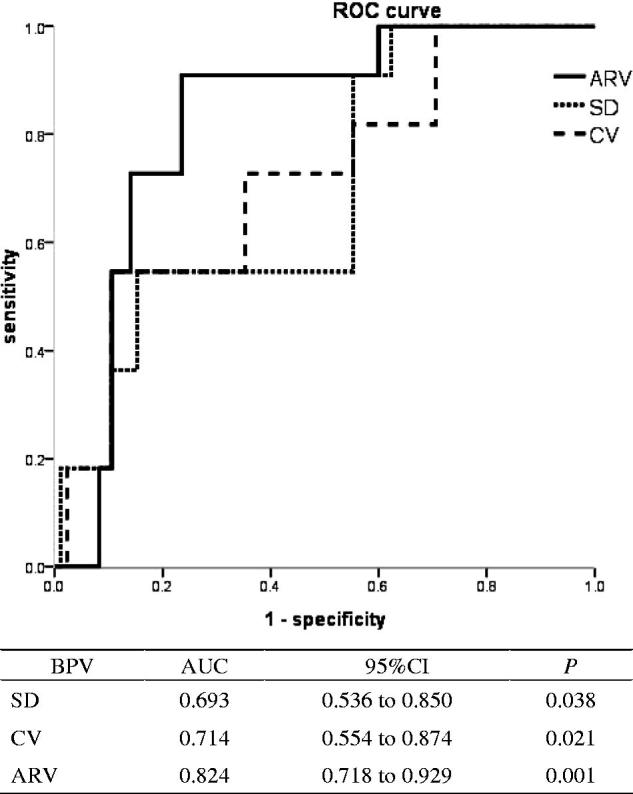
ROC curves for cardiovascular mortality with different blood pressure variability. BPV: blood pressure variability; CV: coefficient of variation; ARV: average real variability.

ROC analyses showed that ARV but not *SD* or CV can accurately predict cardiovascular death in HD patients, with AUC values of 0.824 (*p* = .001), 0.714 (*p* = .021), and 0.693 (*p* = .038), respectively (Figure 4).

### ARV and cohort characteristics

Demographic, clinical, and biochemical characteristics across median of ARV are listed in Table 4. Patients with higher ARV were more likely to be older (*p* = .002); have higher prevalence of diabetes (*p* = .006), larger body mass index (*p* < .01), larger interdialytic weight gain (IDWG; *p* = .004), and higher heart rates (*p* = .033). There was no association of ARV with gender, hemoglobin, albumin, serum calcium, serum phosphate, hs-CRP, ferritin, and parathyroid hormone (Table 3).

**Table 3.** Characteristics of study cohort by median of ARV.

Parameters	Low ARV (*n* = 57)	High ARV (*n* = 46)	*p*
Age (years)	53.89 ± 10.01	61.93 ± 14.44	.002
Sex (% men)	32 (56.14)	28 (60.87)	.628*
Body mass index (kg/m^2^)	21.28 (19.05, 24.02)	24.32 (22.35, 25.95)	<.01^+^
Hemodialysis vintage (months)	64.32 ± 47.73	73.43 ± 35.63	.285
History of diabetes (%)	7 (12.28)	16 (34.78)	.006*
Heart rates (bpm)	72.03 ± 7.20	75.35 ± 8.39	.033
IDWG (kg)	2.28 ± 0.92	2.83 ± 0.95	.004
Hemoglobin (g/L)	121.98 ± 10.58	124.98 ± 13.04	.201
Albumin (g/L)	36.54 ± 2.73	36.18 ± 3.02	.527
Calcium (mmol/L)	2.27 ± 0.18	2.22 ± 0.17	.139
Phosphorous (mmol/L)	1.84 ± 0.56	1.79 ± 0.51	.677
Triglyceride (mmol/L)	1.79 ± 1.10	2.11 ± 1.02	.134
Total cholesterol (mmol/L)	4.43 ± 0.93	4.44 ± 0.92	.906
Ferritin (ng/mL)	614.38 ± 338.49	658.96 ± 403.95	.544
hs-CRP (mg/L)	1.82 (0.89, 2.55)	2.20 (1.61, 6.92)	.057^+^

*p with χ^2^ test and ^+^*p* with rank-sum test.

Hs-CRP: high-sensitivity C-reactive protein; IDWG: interdialytic weight gain; ARV: average real variability.

**Table 4.** Related factors for ARV using logistic regression analysis.

	Multivariate adjusted^a^		
Parameters	*B*	OR (95%CI)	*p*
Age	0.065	1.067 (1.018–1.117)	.005
IDWG	0.825	2.281 (1.416–4.321)	.003

Values expressed as odds ratio and 95%confidence interval (CI).

IDWG: interdialytic weight gain.

^a^Multivariate model: adjusted for age, sex, diabetes mellitus, serum calcium, serum phosphate, hs-CRP, parathyroid hormone and interdialytic weight gain.

### Related factors for ARV

Age and IDWG were positively associated with ARV in the multivariate stepwise logistic analysis (Table 4).

## Discussion

Dialysis patients are routinely exposed to nonphysiologic fluid and osmolar shifts during the dialytic procedure that, combined with impaired counter-regulatory responses, promote more prominent BP changes than are encountered in almost any other clinical circumstance. BPV is categorized as either long- or short term, based on the time interval over which it is considered. Short-term BPV is usually measured by ambulatory BP monitoring during specified short-time intervals. 44-h interdialytic ambulatory BP measurement is the most commonly used method, because of both an increased variability and the gradual increase in BP between dialysis [10,11].

BP measurements for variability studies are commonly quantified as *SD* and CV. Pitfalls of *SD* as an index of BPV have been recently remarked [9]. It only reflects the dispersion of BP measurements around a single value (the mean), not accounting for the order in which the BP measurements were obtained, and is sensitive to the relatively low sampling frequency of noninvasive BP monitoring [9]. An alternative metric to *SD* is the CV, calculated as the ratio of *SD* to the mean. As this metric is based on *SD*, it may also correlate with ambient BP levels, making it suboptimal for describing fluctuation [12].

As an alternative, a new index named ARV has been proposed, which is an average of the absolute differences of consecutive measurements. ARV is sensitive to the individual BP measurement order and less sensitive to the relatively low sampling frequency of noninvasive monitoring. It is a more appropriate index of BPV better describing the injury of additional intermittent stress on the cardiovascular system. It has been reported that intermittent BP load on cardiovascular structures may be as important as tonic BP load, and ARV seems to better describe this phenomenon [13]. Moreover, in contrast to *SD*, ARV could be less obscured by other factors.

Mena et al. [9] using the ARV index showed a statistically significant relative risk (RR: 4.548; 95% CI: 1.53–13.52, *p* = .018) for the group with high 24-h ambulatory BPV with respect to the low BPV group (reference level) in general population; in contrast, the corresponding relative risk associated with the *SD* index was not statistically significant. Hansen et al. [14] showed higher systolic ARV from BP recordings predicted cardiovascular mortality in a large population cohort study (HR: 1.16; 95% CI: 1.07–1.28; *p* < .05). However, to the best of our knowledge, no prior study has examined the association between ARV and cardiovascular mortality in HD population.

In the present study, we found that greater ARV was significantly associated with higher risk of cardiovascular mortality in HD population, even after adjustment for SBP. In contrast, the use of *SD* or CV as a variability index did not confirm this association. It can be hypothesized that ARV is more stable with sampling frequency of BP measurement, and it takes into account the sequential order of BP changes between consecutive readings, better describing the variation of BP.

Studies implementing ABPM have shown that higher BPV is predictive for cardiovascular events and cardiovascular mortality independently of mean BP levels [14–16]. Similar results from the present study show that higher ARV was independently associated with cardiovascular mortality even after adjustment for 44-h SBP.

In this study, we found that age and interdialytic weight gain (IDWG) were associated with greater BP variability in incident HD patients. Along with the age growth, indices related to parasympathetic activity, such as cardiovagal baroreflex sensitivity (BRS) decrease. The decreased BRS in elderly people is associated with increased BPV, bringing additional evidence of cardiovascular autonomic impairment with aging.

Agarwal [17] and Agarwal and Light [18] suggested that IDWG is associated with interdialytic increase (linear trend) in BP, and is related to interdialytic BPV (day-and-night BP rhythm), which indicated that plasma volume changes are the major determinants of BPV. In the present study, ARV as an index of BPV during interdialysis duration was positively related to IDWG. Treatment of essential hypertension with thiazide diuretics in patients on HD supported that volume contraction is a central component that triggers the antihypertensive effect of these drugs. Future studies are needed to examine the important role of volume removal in the modulation of BPV in dialysis patients for prognostic significance.

Several limitations of this study should be recognized. First, the number of subjects enrolled into the study was relatively small, the sample size may not enough to make the conclusion that *SD* and CV of BP had no significant effect on the cardiovascular mortality in HD patients, there is potential for bias in our findings. Second, patients with history of cardiovascular disease were excluded, the result may not be generalized to all the HD patients, further studies are needed to validate the findings. Additionally, in this study, dry weight was not assessed using objective indicator like plasma volume monitoring or bioimpedance analysis, etc. It could not be excluded that individuals with greater BPV were in a state of volume overload. Finally, we adjusted analyses for factors that we believed to be plausibly related to both BPV and mortality. However, we cannot dismiss the potential for other unmeasured confounders.

In conclusion, this study demonstrated that ARV, as an index of BPV, was independently associated with an increased risk of cardiovascular mortality in HD patients, prior to *SD* or CV. Age and IDWG were independent related factors for ARV. Further studies are needed to better understand pathophysiology of BPV and to elucidate the potential therapeutic strategies on BPV to improve cardiovascular outcomes among dialysis patients.

## References

[1] Weiner DE, Tighiouart H, Amin MG, et al Chronic kidney disease as a risk factor for cardiovascular disease and all-cause mortality: a pooled analysis of community-based studies. J Am Soc Nephrol. 2004;15:1307–1315. 15100371 10.1097/01.asn.0000123691.46138.e2

[2] Longenecker JC, Coresh J, Powe NR, et al Traditional cardiovascular disease risk factors in dialysis patients compared with the general population: the CHOICE Study. J Am Soc Nephrol. 2002;13:1918–1927. 12089389 10.1097/01.asn.0000019641.41496.1e

[3] Saran R, Li Y, Robinson B, et al US Renal Data System 2014 Annual Data Report: epidemiology of kidney disease in the United States. Am J Kidney Dis. 2015;66:S1–S305. 10.1053/j.ajkd.2015.05.001 PMC6643986 26111994

[4] Parfrey PS, Foley RN. The clinical epidemiology of cardiac disease in chronic renal failure. J Am Soc Nephrol. 1999;10:1606–1615. 10405218 10.1681/ASN.V1071606

[5] Muntner P, Shimbo D, Tonelli M, et al The relationship between visit-to-visit variability in systolic blood pressure and all-cause mortality in the general population: findings from NHANES III, 1988 to 1994. Hypertension. 2011;57:160–166. 21200000 10.1161/HYPERTENSIONAHA.110.162255

[6] Hsieh YT, Tu ST, Cho TJ, et al Visit-to-visit variability in blood pressure strongly predicts all-cause mortality in patients with type 2 diabetes: a 5-year prospective analysis. Eur J Clin Invest. 2012;42:245–253. 21815887 10.1111/j.1365-2362.2011.02574.x

[7] Gosmanova EO, Mikkelsen MK, Molnar MZ, et al Association of systolic blood pressure variability with mortality, coronary heart disease, stroke, and renal disease. J Am Coll Cardiol. 2016;68:1375–1386. 27659458 10.1016/j.jacc.2016.06.054 PMC5117818

[8] Agarwal R. Blood pressure and mortality among hemodialysis patients. Hypertension. 2010;55:762–768. 20083728 10.1161/HYPERTENSIONAHA.109.144899 PMC2825286

[9] Mena L, Pintos S, Queipo NV, et al A reliable index for the prognostic significance of blood pressure variability. J Hypertens. 2005;23:505–511. 15716690 10.1097/01.hjh.0000160205.81652.5a

[10] Kelley K, Light RP, Agarwal R. Trended cosinor change model for analyzing hemodynamic rhythm patterns in hemodialysis patients. Hypertension. 2007;50:143–150. 17515445 10.1161/HYPERTENSIONAHA.107.091579

[11] Agarwal R. Hypertension diagnosis and prognosis in chronic kidney disease with out-of-office blood pressure monitoring. Curr Opin Nephrol Hypertens. 2006;15:309–313. 16609300 10.1097/01.mnh.0000222700.14960.18

[12] Flythe JE, Brunelli SM. Blood pressure variability among chronic dialysis patients: recent advances in knowledge. Curr Opin Nephrol Hypertens. 2015;24:163–169. 25636142 10.1097/MNH.0000000000000107

[13] Pierdomenico SD, Di NM, Esposito AL, et al Prognostic value of different indices of blood pressure variability in hypertensive patients. Am J Hypertens. 2009;22:842–847. 19498342 10.1038/ajh.2009.103

[14] Hansen TW, Thijs L, Li Y, et al Prognostic value of reading-to-reading blood pressure variability over 24 hours in 8938 subjects from 11 populations. Hypertension. 2010;55:1049–1057. 20212270 10.1161/HYPERTENSIONAHA.109.140798

[15] Mancia G, Bombelli M, Facchetti R, et al Long-term prognostic value of blood pressure variability in the general population: results of the Pressioni Arteriose Monitorate e Loro Associazioni Study. Hypertension. 2007;49:1265–1270. 17452502 10.1161/HYPERTENSIONAHA.107.088708

[16] Stolarz-Skrzypek K, Thijs L, Richart T, et al Blood pressure variability in relation to outcome in the International Database of Ambulatory blood pressure in relation to cardiovascular outcome. Hypertens Res. 2010;33:757–766. 20613762 10.1038/hr.2010.110

[17] Agarwal R. Volume-associated ambulatory blood pressure patterns in hemodialysis patients. Hypertension. 2009;54:241–247. 19528362 10.1161/HYPERTENSIONAHA.109.136366 PMC2745712

[18] Agarwal R, Light RP. Arterial stiffness and interdialytic weight gain influence ambulatory blood pressure patterns in hemodialysis patients. Am J Physiol Renal Physiol. 2008;294:F303–F308. 18160623 10.1152/ajprenal.00575.2007

